# A Case of Rectal Cancer Discovered Following the Occurrence of a Stent-Graft Infection with *Streptococcus gallolyticus* after Thoracic Endovascular Aortic Repair

**DOI:** 10.3400/avd.cr.25-00047

**Published:** 2025-09-12

**Authors:** Koki Yokawa, Taku Nakagawa, Makoto Kusakizako, Yosuke Tanaka, Tomonori Higuma, Kazunori Yoshida, Yoshihiro Oshima, Hidefumi Obo, Hidetaka Wakiyama

**Affiliations:** Department of Cardiovascular Surgery, Kakogawa Central City Hospital, Kakogawa, Hyogo, Japan

**Keywords:** rectal cancer, thoracic endovascular aortic repair, stent-graft infection

## Abstract

A 79-year-old man was admitted for transurethral resection of a bladder cancer. He had a history of thoracic endovascular aortic repair for Stanford type B acute aortic dissection and thoracic aortic aneurysm performed 2 years prior. During hospitalization, computed tomography scan findings raised suspicion of a stent-graft infection. Blood cultures confirmed the presence of *Streptococcus gallolyticus* ssp. *pasteurianus*. Gallium scintigraphy supported the diagnosis of a stent-graft infection. A subsequent lower gastrointestinal endoscopy revealed a colorectal cancer in the lower rectum. We then performed surgery for the stent-graft infection.

## Introduction

It has been reported that 25%–80% of patients with *Streptococcus gallolyticus* bacteremia have concomitant colorectal tumors. Colonic neoplasia may arise years after the presentation of bacteremia or infectious endocarditis due to *S. gallolyticus*. In the present report, we describe a case of rectal cancer discovered after the occurrence of a stent-graft infection caused by *S. gallolyticus*.

## Case Report

**Fig. 1:**
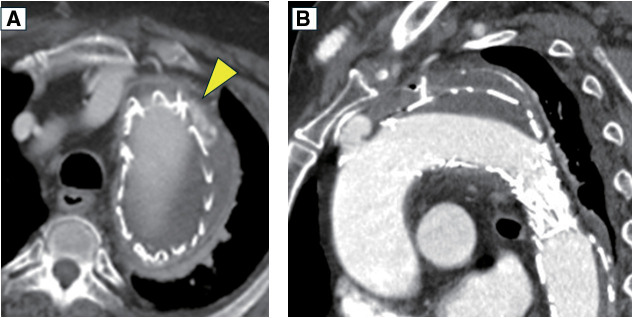
(**A**) Computed tomography scan findings (yellow arrowhead) showing minor type Ia endoleak. (**B**) Computed tomography scan findings raising suspicion of an infected thoracic aortic aneurysm.

**Fig. 2:**
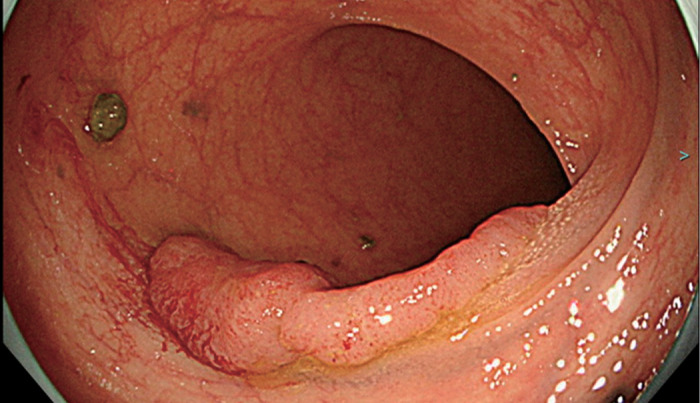
Lower gastrointestinal endoscopy revealing a rectal cancer.

**Fig. 3:**
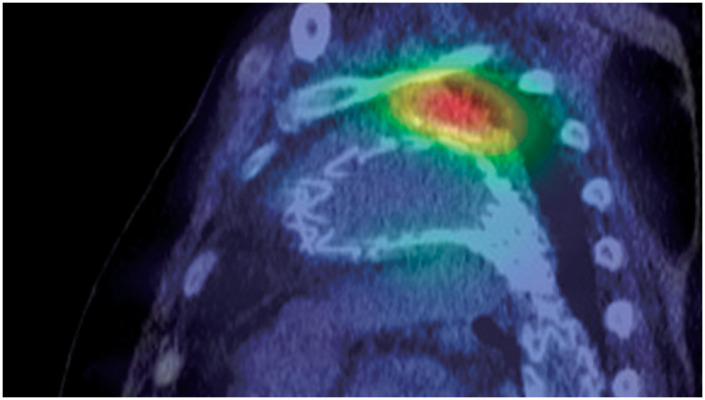
Preoperative gallium scintigraphy findings indicating the presence of an infected thoracic aortic aneurysm and stent-graft infection.

A 79-year-old man had previously undergone 2-debranched thoracic endovascular aortic repair using 2 cTAG (W. L. Gore & Associates, Flagstaff, AZ, USA) with bypasses from the right axillary artery to the left common carotid artery and the left axillary artery 2 years ago due to a 53-mm distal aortic arch aneurysm complicated by Stanford type B acute aortic dissection. Since then, his aortic diameter has remained unchanged, and the patient has been stable. Four years prior, the patient had undergone transurethral resection of bladder tumor (TURBT) for bladder cancer, and 2 additional resections were performed due to suspected recurrence. The patient was then readmitted for TURBT. Prior to admission, he had a fever with a temperature of approximately 37.5°C. Initial blood tests revealed a white blood cell count of 7900/μL and a C-reactive protein level of 20 mg/dL, prompting a CT scan for further evaluation. The CT scan showed minor type Ia endoleak and wall thickening of the thoracic aortic aneurysm, raising suspicion of a stent-graft infection (**Fig. 1**). Blood cultures identified *S. gallolyticus* ssp. *pasteurianus*, requiring antibiotic therapy. Ceftriaxone 2 g once daily was administered for 3 days. After identification of the causative organism by blood culture, ampicillin 2 g three times daily was continued for approximately 3 weeks until surgery. Although the fever subsided following the initiation of antibiotic therapy, C-reactive protein (CRP) levels did not normalize; the lowest recorded value was 4.8 mg/dL, indicating persistent inflammation. Given that this bacterium is known to be associated with colorectal cancer, a lower gastrointestinal endoscopy was performed, which confirmed the presence of a rectal cancer (**Fig. 2**). The tumor was located in the lower rectum (Rb) and was classified as type 1 (protruding type) cancer. The predicted depth of invasion was into the muscularis propria. A biopsy was performed, and the pathological diagnosis was adenocarcinoma, tub1, tub2, group 5. For further assessment of the infection, gallium scintigraphy was conducted, which showed tracer accumulation in the distal aortic arch aneurysm and stent-graft, suggestive of an infected thoracic aortic aneurysm and stent-graft infection (**Fig. 3**). The patient continued with the antibiotic treatment, and as his condition stabilized, rectal cancer surgery was initially planned. However, the patient developed back pain, and imaging revealed an enlargement of the distal aortic arch aneurysm. At the time, the patient reported back pain, and the CRP level had risen again to 17 mg/dL. Consequently, the treatment strategy was revised to prioritize surgery for the infected thoracic aortic aneurysm and stent-graft infection. The procedure was performed via median sternotomy and involved total aortic arch replacement and partial removal of the infected stent-graft. The aortic aneurysm was successfully resected, and the intraluminal thrombus was removed and submitted for culture; however, the culture results were negative. Intraoperatively, bleeding control at the distal anastomosis site proved challenging; thus, the surgery was completed with the patient’s chest left open. The operation time was 11 hrs and 16 min, with a cardiopulmonary bypass time of 507 min, a myocardial ischemic time of 229 min, and a lower body circulatory arrest time of 165 min. Postoperatively, he experienced severe cardiac dysfunction, requiring circulatory support. Despite continued intensive care treatments, the patient died on postoperative day 18.

## Discussion

It has been reported that 25%–80% of patients with *S. gallolyticus* bacteremia have concomitant colorectal tumors.^1)^ Moreover, 94% of *Streptococcus bovis* bacteremia cases associated with colorectal cancer were in fact *S. bovis* biotype I cases, whereas only 18% were biotype II cases.^2)^ *S. bovis* biotype II/2 was renamed as *S. gallolyticus* subspecies *pasteurianus* and *S. gallolyticus* subspecies *macedonicus. S. bovis*/*gallolyticus* bacteria, especially their cell wall antigens, were found to remarkably increase the production of inflammatory cytokines in the colonic mucosa of rats, specifically the inflammation-mediated pathway involving interleukin-1 (IL-1), cyclooxygenase-2, and IL-8,^3)^ suggesting direct interaction between *S. bovis* and colonic mucosal cells, which is thought to lead to the development of colorectal cancer.

Although McCoy and Mason^4)^ have suggested a relationship between colonic carcinoma and the presence of infectious endocarditis in 1951, it was only in 1974 that the association between *S. bovis* and colorectal neoplasia was recognized.^5)^ Therefore, in cases of *S. gallolyticus* bacteremia, clinicians should consider the possibility of an underlying malignancy and conduct appropriate diagnostic investigations on the patients.

Regarding the surgical approach, both the infected thoracic aortic aneurysm and the stent-graft infection required an intervention. In the present case, a median sternotomy was chosen as the surgical approach. However, for the comprehensive treatment of both conditions, an anterolateral thoracotomy with partial sternotomy (ALPS) might have been more appropriate. Nonetheless, considering the patient’s advanced age, the simultaneous performance of sternotomy and left thoracotomy was deemed excessively invasive. Yamanaka et al. have suggested that ALPS is an effective surgical approach for stent-graft infections.^6)^ If ALPS had been employed, better control of bleeding at the distal anastomotic site could have been achieved, potentially leading to a more successful completion of the surgery.

## Conclusion

The present case highlights the importance of considering underlying malignancies in patients presenting with graft infections caused by *S. gallolyticus*, thereby illustrating the association between graft infections and colorectal malignancy.
